# Pleural Calcified Tumor Misdiagnosed as Peripheral Lung Cancer: A Case Report and Analysis of Imaging Differential Points

**DOI:** 10.1002/ccr3.72879

**Published:** 2026-06-18

**Authors:** Tengfang Zhang, Yilian Xie, Hao Chen, Qingyong Cai, Baolei liang, Zhengguo Wu

**Affiliations:** ^1^ Department of Cardiothoracic Surgery The Fifth Affiliated Hospital of Southern Medical University Guangzhou Guangdong China; ^2^ Department of Cardiothoracic Surgery Yantian District People's Hospital Shenzhen Guangdong China

**Keywords:** case report, differential diagnosis, misdiagnose, peripheral lung cancer, pleural calcified solitary fibroma (calcified SFT)

## Abstract

Pleural calcified solitary fibrous tumor, though rare, should be considered in the differential diagnosis of peripheral lung nodules. Recognizing key imaging features such as broad‐based pleural attachment and coarse calcification can prevent misdiagnosis and avoid unnecessary lung resection, thereby preserving pulmonary function and improving patient outcomes.

## Graphical abstract

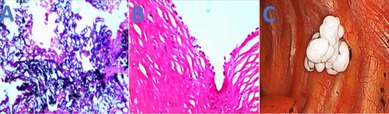

**Abbreviations**
CFT: calcifying fibrous tumor
CT: computed tomography
EUS: endoscopic ultrasonography
FDG‐PET: fluorodeoxyglucose positron emission tomography
MRI: Magnetic Resonance Imaging
SFT: solitary fibrous tumor

## 1 Introduction

Lung cancer is one of the malignant tumors with the highest morbidity and mortality in the world, which constitutes a major public health challenge. According to global cancer statistics, there are more than 2.2 million new cases and 1.8 million deaths of lung cancer every year, and its 5‐year survival rate is generally low, which is closely related to late diagnosis [1]. This severe epidemiological situation highlights the extreme importance of early diagnosis in improving the prognosis and survival rate of patients. CT screening in high‐risk groups (such as long‐term smokers) has been confirmed by many large‐scale randomized controlled trials (such as NLST and NELSON studies) to reduce the specific mortality of lung cancer by more than 20% [2]. Therefore, CT has become the gold standard imaging tool for the detection and qualitative diagnosis of pulmonary nodules, and its high spatial resolution can clearly display the shape, density, edge characteristics and dynamic changes of nodules, providing a key basis for clinical decision‐making. However, although CT technology is getting better and better, its diagnosis is not perfect and there is a certain misdiagnosis rate. This limitation mainly comes from the following aspects: First, some benign lesions, such as inflammatory pseudotumor, tuberculoma, organized pneumonia and some benign pleural tumors (such as solitary fibroma of pleura (calcified SFT)), may show signs similar to malignant tumors (such as lobulation, burr and pleural traction) on CT images, thus leading to false positive diagnosis [3]. Secondly, some early lung cancer with inert growth or atypical manifestations (such as ground‐glass nodules or some solid nodules) may be misjudged as benign or inflammatory lesions, resulting in false negative results [4]. In addition, the differences in experience of radiologists and the subjectivity in the diagnosis process further aggravate the risk of misjudgment.

Pleural calcifying tumor, namely solitary fibroma of pleura (calcified SFT) with obvious calcification, is an extremely rare mesenchymal tumor. In imaging, the manifestations of SFT are diverse. Usually, it is an isolated mass with a clear boundary originating from the pleura, accompanied by thick and dense calcification inside [5]. This imaging combination of “pleural origin” and “significant calcification” constitutes a significant diagnostic trap in clinical practice because it is easily confused with more common pleural lesions, such as encapsulated effusion, pleural tuberculoma, and malignant mesothelioma.

To sum up, although CT plays a central role in the diagnosis of lung cancer, clinicians still need to be cautious about its diagnostic limitations. For lesions with atypical imaging manifestations, especially those adjacent to pleura or mediastinum, we should keep a high degree of vigilance and actively use multimodal imaging technology or pathological examination to make comprehensive discrimination, so as to optimize the diagnosis and treatment strategy and minimize the risk of misdiagnosis. This study reported a case of pleural calcified SFT misdiagnosed as peripheral lung cancer before operation, and combined with literature review, aiming at systematically expounding the imaging characteristics of this disease and deeply analyzing the main points of its differentiation from peripheral lung cancer, in order to improve the clinical diagnosis and treatment level of this rare disease [6].

## 2 Case History and Examination

### 2.1 Patient Information and Chief Complaint

A 52‐year‐old male patient was treated for “repeated cough for 4 months”. Physical examination: t 36.2°C, p 92 times/min, R 20 times/min, BP 137/91 mmHg. No positive signs were found. He had a history of hypertension for about 5 years, multiple stones in the left kidney, and fatty liver.

### 2.2 Imaging Examination

**FIGURE 1:**
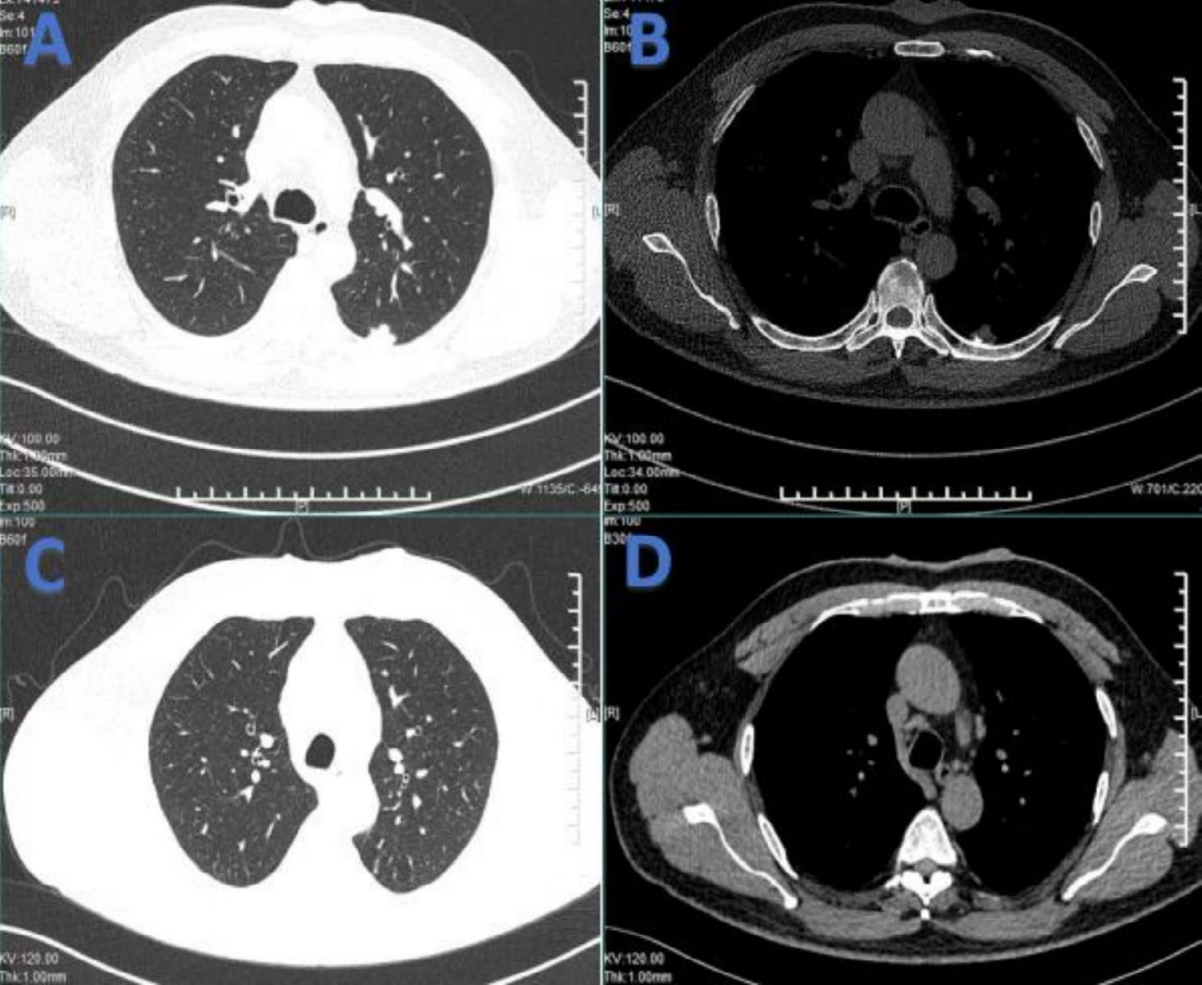
Preoperative (A, B) and postoperative (C, D) chest CT.

Chest CT plain scan showed that a nodular shadow was seen in the posterior segment of the upper tip of the left lung, with a size of about 16 × 11 mm, patchy calcification at the edge and rough bone cortex adjacent to the ribs; there is a cord shadow in the middle lobe of the right lung (considering the old focus). There is no thickening of bilateral pleura, and no pleural effusion is found. Preliminary imaging diagnosis: peripheral lung cancer with calcification (Figure 1).

## 3 Diagnosis and Treatment

### 3.1 Preoperative Preparation and Surgical Decision

After preoperative evaluation and discussion, the initial treatment plan is to perform wedge resection of the left lung tumor under thoracoscope.

### 3.2 Intraoperative Findings

**FIGURE 2:**
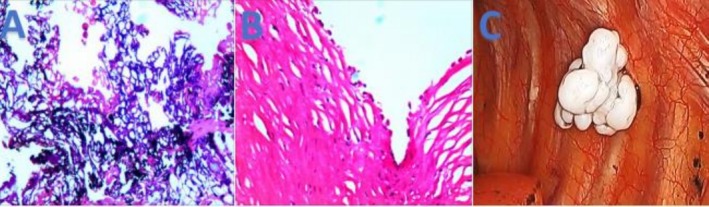
Postoperative pathology (A, B) and thoracic exploration (C). (A) Microscopic view showing abundant basophilic calcified deposits (dark blue–purple areas) within the pleural fibrous tissue, accompanied by reactive hyperplasia of surrounding connective tissue and mild inflammatory cell infiltration. These calcified foci correspond to the calcified shadow observed on preoperative imaging. (B) Microscopic view showing dense, mature collagen fibers (eosinophilic pink areas) and a continuous layer of benign‐appearing mesothelial cells on the pleural surface. No obvious nuclear atypia, malignant cell nests, or glandular structures are identified, confirming the benign nature of the lesion.

During thoracoscopic exploration, there was no abnormality in the left upper lung and other lungs, and a white cauliflower‐like tumor with a size of about 2 cm was seen at the back of the left chest wall about the fourth rib, which was consistent with the position shown by CT (Figure 2). The tumor has no adhesion with lung tissue and is easy to separate. Parietal pleura can see scattered multiple black spots about 0.5 cm in size, and a little effusion in pleural cavity. Intraoperative diagnosis: left chest wall tumor. Therefore, the surgical plan was changed, and the chest wall tumor was completely removed and the left pleural biopsy was performed.

### 3.3 Pathological Results

The tumor in the left chest wall is generally gray–white bony verrucous process. Microscopically, a polypoid tumor was seen, with a low columnar epithelium on the surface and a few spherical structures under it. Special staining was negative for PAS. The final pathological diagnosis: (left chest wall tumor) is consistent with pleural calcifying tumor (solitary fibroma of pleura with significant calcification (calcifying SFT)). The biopsy tissue of left pleura showed no definite diagnostic features due to compression degeneration (Figure 2).

## 4 Conclusion

We reported a case of pleural calcified SFT misdiagnosed as peripheral lung cancer before operation and combined with literature review, aiming at systematically expounding the imaging characteristics of this disease and deeply analyzing the main points of its differentiation from peripheral lung cancer, in order to improve the clinical diagnosis and treatment level of this rare disease.

## 5 Discussion

Pleural calcifying tumor is a rare mesenchymal subtype of solitary fibrous tumor (SFT) characterized by prominent dystrophic calcification, accounting for < 5% of all pleural tumors [5]. It originates from dendritic interstitial cells rather than mesothelial cells; thus, the old term “localized mesothelioma” has been abandoned. The tumor occurs at any age, with a peak incidence between 50 and 70 years and no gender predilection [7]. Most SFTs are benign (≈80%), but 10%–20% exhibit malignant or potentially malignant behavior [8]. Unlike malignant mesothelioma, pleural SFT shows no clear association with asbestos exposure. Grossly, SFT is typically a solitary, encapsulated, lobulated mass with a gray‐white, firm cut surface; larger tumors may show hemorrhage, cystic degeneration, or necrosis. The calcified variant displays visible gritty calcifications. Histopathologically, SFT demonstrates a classic “patternless pattern” with alternating hypercellular and hypocellular areas (spindle cells and collagen‐rich stroma) and characteristic staghorn vessels [9]. Immunohistochemistry is key for diagnosis: SFT cells diffusely express CD34 (> 90%), Bcl‐2, and STAT6. Nuclear positivity for STAT6 is the most specific marker, reflecting the NAB2‐STAT6 gene fusion [10]. Histologically, calcification is dystrophic calcification, which is more common in tumors with long course and slow growth. Imaging findings of SFT are diverse, which is closely related to its pathological components. Pleural calcified tumor is a rare pleural tumor. Its diagnosis depends on the imaging findings of a mass closely related to the chest wall, obviously enhanced and possibly accompanied by calcification, combined with postoperative pathological and characteristic immunohistochemical markers (especially STAT6) to make the final diagnosis. Understanding its epidemiological, pathological and imaging features is helpful to distinguish it from more common peripheral lung cancer in clinical practice.

We performed a literature search (January 1996–December 2024) in PubMed using the keywords “calcified SFT” and “pleura.” Table 1 summarizes 43 reported cases, including ours. The mean age was 35 years (range 7–68), with only one patient > 65 years. Calcified SFT was more common in young women (male: female = 17:26). Twenty‐seven patients had multiple lesions and 16 solitary lesions; 23 were symptomatic and 20 asymptomatic. Our case (a 52‐year‐old male) suggests a broader disease spectrum. Based on surgical completeness, we divided patients into complete (*n* = 30) and incomplete (*n* = 12, including one without surgery) resection groups (Table 2). No significant differences were observed in age (*p* = 0.71), sex (*p* = 0.50), or symptoms (*p* = 0.96). However, multiple lesions were significantly associated with incomplete resection (*p* = 0.03), indicating greater surgical difficulty for multifocal disease. Table 3 compares the imaging features of calcified SFT and peripheral lung cancer. Analysis of our misdiagnosis revealed two key oversights: first, the wide‐base pleural attachment was not recognized preoperatively; second, internal calcification was misinterpreted as a rare manifestation of lung cancer, without considering that SFT is a common cause of calcification in chest wall tumors.

**TABLE 1:** Reported cases of pleural calcifying fibrous tumor.

No.	Author	Year	Age	Gender	Focality	Symptoms	Surgery
1	Pinkard	1996	23	F	Multiple	Present	Complete
2	Pinkard	1996	28	F	Multiple	Absent	Complete
3	Pinkard	1996	34	M	Solitary	Present	Complete
4	Hainaut	1996	29	F	Multiple	Absent	Incomplete
5	Cavazza	2002	46	F	Solitary	Absent	Complete
6	Ammar	2003	38	F	Solitary	Present	Complete
7	Jang	2004	31	F	Solitary	Absent	Complete
8	Soyer	2004	7	M	Solitary	Present	Complete
9	Mito	2005	54	M	Multiple	Absent	Incomplete
10	Kawhara	2005	35	F	Multiple	Present	Incomplete
11	Yasukawa	2006	35	F	Multiple	Present	Complete
12	Shibata	2008	52	F	Multiple	Absent	Incomplete
13	Suh	2008	35	M	Multiple	Absent	Complete
14	Miyano	2008	44	F	Multiple	Absent	Complete
15	Sleigh	2010	22	F	Multiple	Present	Incomplete
16	Yokosuka	2010	40	F	Solitary	Absent	Complete
17	Isaka	2011	40	M	Multiple	Present	Complete
18	Jiang	2011	44	F	Multiple	Present	Complete
19	Agackiran	2012	40	M	Multiple	Present	Complete
20	Fujita	2012	58	F	Solitary	Absent	Complete
21	Ishida	2013	31	M	Multiple	Absent	Incomplete
22	Azam	2014	31	M	Multiple	Absent	No resection
23	Matsumoto	2014	20	F	Multiple	Absent	Complete
24	Nakagawa [6]	2014	30	F	Multiple	Present	Complete
25	Minerowic	2015	15	F	Multiple	Present	Incomplete
26	Lee	2015	47	F	Solitary	NA	Complete
27	Rocas	2015	59	M	Solitary	Absent	Complete
28	Sawaga	2017	55	F	Multiple	Absent	Incomplete
29	Edlin	2018	23	F	Solitary	Present	Complete
30	Mazi	2018	15	F	Multiple	Present	Complete
31	Lisowsk	2018	27	F	Solitary	Absent	Complete
32	Mehrad	2018	32	M	Multiple	Present	Incomplete
33	Mehrad	2018	21	M	Solitary	Absent	Complete
34	Mehrad	2018	32	F	Multiple	Absent	Complete
35	Massoth	2019	59	M	Multiple	Absent	Incomplete
36	Bono	2020	10	M	Solitary	Present	Complete
37	Miyamoto	2020	21	F	Multiple	Absent	Incomplete
38	Gorai	2020	52	F	Solitary	Absent	Complete
39	Hernandez	2021	35	M	Multiple	Present	Complete
40	Jia	2021	38	M	Multiple	Present	Incomplete
41	RyoYokota	2024	31	F	Solitary	Absent	Complete
42	Fumihiro	2024	68	M	Multiple	Absent	Complete
43	Our case	2025	52	M	Solitary	Present	Complete

*Note:* See Supporting Information for references in this table.

**TABLE 2:** Characteristics of patients with complete or incomplete resection of pleural calcifying fibrous.

Characteristics	Complete resection	Incomplete resection	*p*
All cases	30	12	
Age at diagnosis of tumor (years)			
Median	35.5	35	0.54
Range	10–68	15–59	
Gender, *n* (%)			
Male	10 (33.3%)	5 (41.7%)	0.74
Female	20 (66.7%)	7 (58.3%)	
Symptoms, *n* (%)			
Present	14 (46.7%)	7 (58.3%)	0.72
Absent	16 (53.3%)	5 (41.7%)	
Characteristics of lesions, *n* (%)			
Solitary	17 (56.7%)	2 (16.7%)	0.03
Multiple	13 (43.3%)	10 (83.3%)	

*Note:* The statistical methods used: continuous variables (age) were compared using the Mann–Whitney *U* test; categorical variables were compared using Fisher's exact test due to small theoretical frequencies.

**TABLE 3:** Key imaging features for differentiating calcified solitary fibrous tumor of the pleura from peripheral lung cancer.

Feature	Calcified solitary fibrous tumor of the pleura (calcifying SFT)	Peripheral lung cancer
Relationship with Chest Wall	Broad‐based, contiguous attachment; presence of a pleural tail sign/pleural thickening.	May abut the pleura but typically forms an acute angle; absence of a pleural tail sign.
Calcification Pattern	Common; irregular, patchy, diffuse, and extensive.	Uncommon; typically eccentric, stippled, or fine granular with limited extent.
Enhancement Pattern	Mild to moderate heterogeneous enhancement (moderate vascularity).	Moderate to marked enhancement with diverse patterns (rim‐like, heterogeneous, homogeneous); hypervascular.
Morphology & Margin	Round or oval‐shaped with well‐defined, smooth margins.	Irregular shape with lobulated or spiculated margins, ill‐defined borders.
Internal Density/Signal	Heterogeneous density; high density in calcified areas with intermediate density elsewhere.	Heterogeneous density/signal; may show bubble lucencies, air bronchograms, or necrotic/cystic changes.
Growth Pattern	Predominantly grows towards the chest wall, displacing adjacent structures.	Predominantly grows into the lung parenchyma, infiltrating surrounding tissue.
Associated Findings	Usually no hilar/mediastinal lymphadenopathy or pleural effusion.	May be associated with hilar/mediastinal lymphadenopathy, pleural effusion, or pleural metastases.

Although chest CT (Computed Tomography) is the first choice for the initial imaging examination to evaluate pleural tumors, MRI (Magnetic Resonance Imaging) and PET‐CT (Fluorodeoxyglucose Positron Emission Tomography) play a crucial auxiliary role in the qualitative diagnosis, preoperative planning and malignant risk assessment of pleural calcifications. SFT usually shows a characteristic “black and white” or “paving stone” signal pattern on MRI. The multiplanar imaging ability of MRI makes it superior to CT in evaluating the relationship between huge or complex SFT and surrounding structures (such as diaphragm, spinal canal and mediastinal vessels). This is very important for making an accurate surgical plan and predicting the difficulty and risk of surgery [11]. FDG‐PET‐CT provides a unique perspective for the differential diagnosis and systemic evaluation of SFT by displaying the glucose metabolic activity of tumors. To sum up, MRI has advantages in showing the relationship between tumor and chest wall and internal structure. On PET‐CT, SFT can show hypermetabolism, which is easily confused with lung cancer.

**FIGURE 3:**
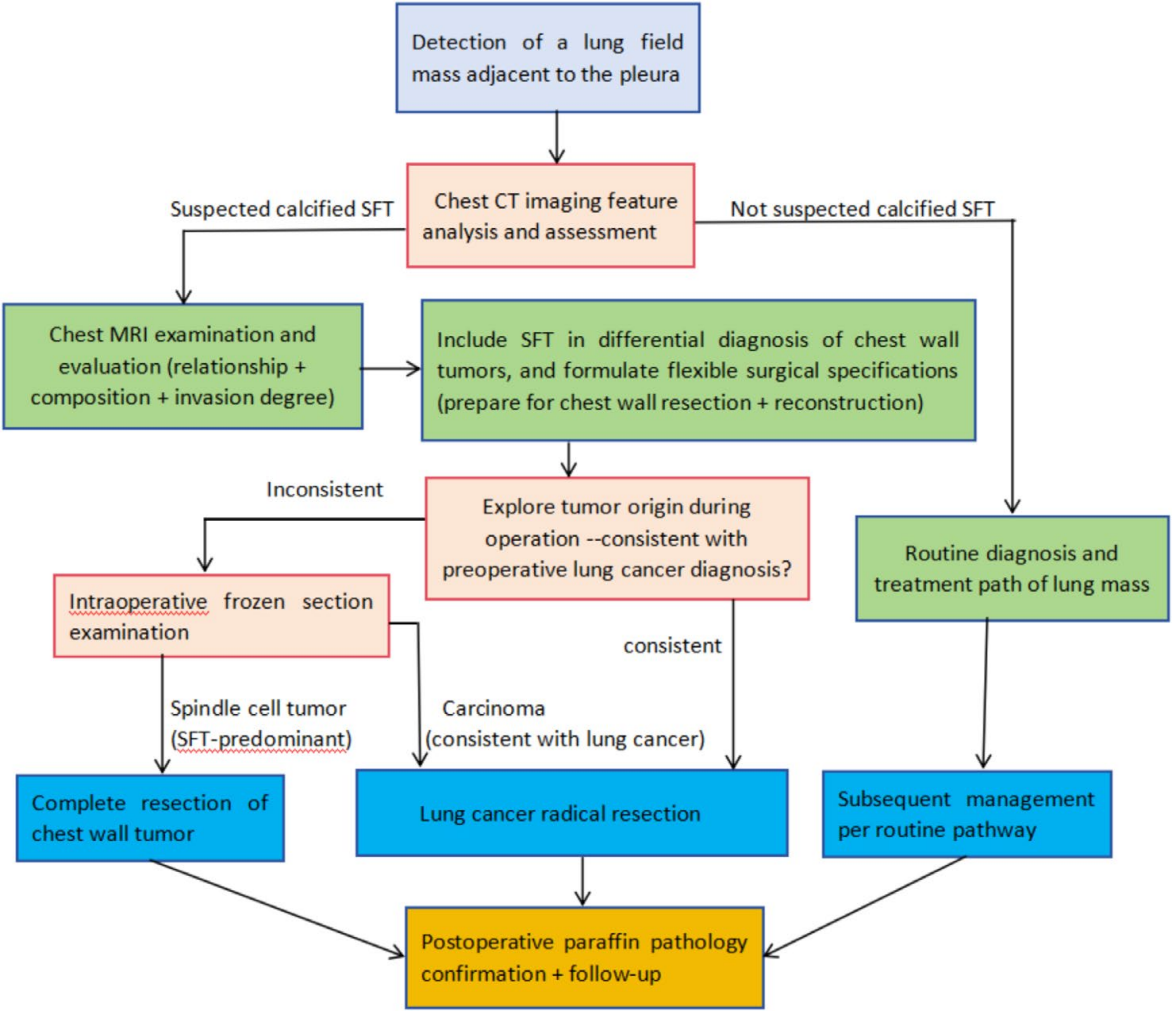
Standardized diagnosis and treatment flow chart.

Based on this case, we propose a standardized diagnostic and treatment algorithm (Figure 3). Initial evaluation should focus on chest CT features: broad‐based pleural attachment and gross calcification, suggesting pleural origin [12]. When calcified SFT is suspected, MRI is recommended to better delineate tumor‐chest wall relationships and internal composition, aiding differential diagnosis and surgical planning [6]. SFT should be formally included in the differential diagnosis of chest wall tumors. Surgical planning must be flexible, prepared for chest wall resection and reconstruction. Intraoperative frozen section is critical: when exploration reveals a chest wall origin inconsistent with preoperative suspicion of lung cancer, frozen section can reliably distinguish spindle cell tumors (e.g., SFT) from carcinoma, redirecting surgery from lung resection to complete chest wall tumor excision, thereby avoiding unnecessary lobectomy and preserving healthy lung tissue [7].

The core lesson of this case is that for the so‐called “intrapulmonary” nodules that are close to the pleura in imaging, clinicians and radiologists must break through the inertia thinking, broaden the vision of differential diagnosis, and take calcified chest wall‐derived tumors such as SFT into routine consideration. The final diagnosis and treatment decision should be based on meticulous multidisciplinary cooperation: through accurate interpretation of image details by the radiology department, careful planning of the surgical plan by the thoracic surgery department, and final diagnosis of specimens by the pathology department, a complete diagnosis and treatment chain will be formed together. Only through this systematic and multidisciplinary evaluation model and continuous improvement of the understanding of rare diseases such as pleural SFT can we avoid misdiagnosis to the greatest extent and finally ensure that every patient can receive the most appropriate and individualized treatment.

## Author Contributions

**Tengfang Zhang:** data curation, visualization, writing – original draft. **Yilian Xie:** data curation, visualization, writing – original draft. **Hao Chen:** data curation, investigation, writing – review and editing. **Qingyong Cai:** data curation, supervision. **Baolei liang:** data curation, investigation, writing – review and editing. **Zhengguo Wu:** conceptualization, visualization, writing – review and editing.

## Funding

The present study was supported in part by the Dean's Fund of the Fifth Affiliated Hospital of Southern Medical University (grant no. YZ2023ZX09) and Shenzhen Yantian District Medical and Health Science and Technology Plan Project (YTWS20220108).

## Ethics Statement

The authors have nothing to report.

## Consent

Written informed consent was obtained from the patient.

## Conflicts of Interest

The authors declare no conflicts of interest.

## Supporting information


**Data S1:** References for Table 1.

## Data Availability

The datasets used during the current study are available from the corresponding author upon reasonable request.
