# Supplementary material for: Pleural Calcified Tumor Misdiagnosed as Peripheral Lung Cancer: A Case Report and Analysis of Imaging Differential Points
Source: Clin Case Rep. 2026 Jun 18;14(6):e72879. doi: 10.1002/ccr3.72879 (PMC13279434; doi:10.1002/ccr3.72879)
Supplement: Supplementary file 1 — Data S1: References for Table 1. [file CCR3-14-e72879-s001.docx]

Supplementary Material: References for Table 1

The following references correspond to the 43 reported cases of pleural calcifying fibrous tumor (calcified SFT) summarized in Table 1.

1.Pinkard NB, et al. (1996) – See reference below for three cases (#1-3).

Pinkard NB, et al. Solitary fibrous tumor of the pleura: a clinicopathologic review of 23 cases. Am J Surg Pathol. 1996;20(12):1468-1477.

2.Hainaut P, et al. (1996)

Hainaut P, Lesage V, Weynand B, Coche E. Calcifying fibrous pseudotumor of pleura: a case report of three cases of a newly described entity involving the pleura. Am J Clin Pathol. 1996;105(2):189-194.

3.Cavazza A, et al. (2002)

Cavazza A, Gelli MC, Agostini L, Sgarbi G, De Marco L, Gardini G. [Calcified pseudotumor of the pleura: description of a case]. Pathologica. 2002;94(4):201-205. doi:10.1007/s102420200032. PMID: 12325419.

4.Ammar A, et al. (2003)

Ammar A, El Hammami S, Horchani H, Sellami N, Kilani T. Calcifying fibrous pseudotumor of the pleura: a rare location. Ann Thorac Surg. 2003;76(6):2081-2082. doi:10.1016/S0003-4975(03)00741-0.

5.Jang KY, et al. (2004)

Jang KS, Oh YH, Han HX, Chon SH, Chung WS, Park CK, et al. Calcifying fibrous pseudotumor of the pleura. Ann Thorac Surg. 2004;78(6):e87-e88. doi:10.1016/j.athoracsur.2004.04.028.

6.Soyer T, et al. (2004)Soyer T, Ciftci AO, Güçer S, Orhan D, Senocak ME. Calcifying fibrous pseudotumor of lung: a previously unreported entity. J Pediatr Surg. 2004;39(11):1729-1730. doi:10.1016/j.jpedsurg.2004.05.018.

7.Mito K, et al. (2005)

Mito K, Kashima K, Daa T, Kondoh Y, Miura T, Kawahara K, Nakayama I, Yokoyama S. Multiple calcifying fibrous tumors of the pleura. Virchows Arch. 2005;446(1):78-81. doi:10.1007/s00428-005-0074-4.

8.Kawahara K, et al. (2005)

Kawahara K, Yasukawa M, Nakagawa K, Katsura H, Nagano T, Iwasaki T. Multiple calcifying fibrous tumor of the pleura. Virchows Arch. 2005;447(6):1007-1008. doi:10.1007/s00428-005-0074-4.

9.Yasukawa M, et al. (2006)

Yasukawa M, et al. A case of multiple calcifying fibrous pseudotumor developed in the pleura. J Jpn Assoc Chest Surg. 2006;20(4):677-681.

10.Shibata K, et al. (2008)

Shibata K, et al. Multiple calcifying fibrous pseudotumors disseminated in the pleura. Ann Thorac Surg. 2008;85(2):e10-e11. doi:10.1016/j.athoracsur.2007.10.048.

11.Suh JH, et al. (2008)

Suh JH, Shin OR, Kim YH. Multiple calcifying fibrous pseudotumor of the pleura. J Thorac Oncol. 2008;3(11):1356-1358. doi:10.1097/JTO.0b013e318186a87a. PMID:18978573.

12.Miyano K, et al. (2008)

Miyano K, et al. Multiple calcifying fibrous pseudotumor of the pleura. Ann Thorac Cardiovasc Surg. 2008;14(1):38-41.

13.Sleigh K, et al. (2010)

Sleigh K, et al. Calcifying fibrous pseudotumours: an unusual case with multiple pleural and mediastinal lesions. Interact Cardiovasc Thorac Surg. 2010;10(4):624-626. doi:10.1510/icvts.2009.227918.

14.Yokosuka T, et al. (2010)

Yokosuka T, Kondo K, Ota Y, et al. A case of calcifying fibrous tumor of the pleura treated by VATS. J Jpn Assoc Chest Surg. 2010;24(1):35-38. doi:10.2995/jacsurg.24.35.

15.Isaka T, et al. (2011) – Reference [5] in manuscript.

Isaka T, et al. (2011). [Title in Japanese; see Table 1 - 40‑year‑old male, multiple lesions, symptomatic, complete resection]. J Thorac Dis. 2011;3(4). Abstract.

16.Jiang K, et al. (2011)

Jiang K, Nie J, Wang JJ, Li JS. Multiple calcifying fibrous pseudotumor of the bilateral pleura. Jpn J Clin Oncol. 2011;41(1):130-133. doi:10.1093/jjco/hyq108.

17.Ağaçkıran Y, et al. (2012)

Ağaçkıran Y, Fındık G, Aydoğdu K, Günay E, Günay S, Kaya S. An extremely rare case of multiple calcifying tumor of the pleura. Tuberk Toraks. 2012;60(4):385-388.

18.Fujita S, et al. (2012)

Fujita S, et al. Calcifying fibrous pseudotumor of the diaphragmatic pleura: report of a case. J Jpn Assoc Chest Surg. 2012;26(4):453-458. doi:10.2995/jacsurg.26.453.

19.Ishida M, et al. (2013)

Ishida M, Okabe H, Oka T, et al. Disseminated calcifying tumor of the pleura. Pathol Int. 2013;63(6):333-335. doi:10.1111/pin.12072. PMID:23742055.

20.Azam F, et al. (2014)

Azam F, et al. Multifocal calcifying fibrous tumor at six sites in one patient: a case report. World J Surg Oncol. 2014;12:235. doi:10.1186/1477-7819-12-235.

21.Matsumoto H, et al. (2014)

Matsumoto H, Komori S, Maehara T, Nakamura K, Sano A, Sakai H, Sakao Y. A case of multiple calcifying fibrous tumors of the pleura. Jpn J Chest Surg (J Jpn Assoc Chest Surg). 2014;28(1):44-48. doi:10.2995/jacsurg.28.44.

22.Nakagawa T, et al. (2014) – Reference [6] in manuscript.

Nakagawa M, Hanagiri T, Orihashi T, Yoshii C, Mukae H, Tanaka F. A case of a slowly enlarging solitary fibrous tumor of the pleura. Jpn J Lung Cancer. 2014;54(2):63-67. doi:10.2482/haigan.54.63.

23.Minerowicz C, et al. (2015)

Minerowicz C, Jagpal S, Uppaluri L. Calcifying fibrous pseudotumor of the pleura. Am J Respir Crit Care Med. 2015;191(6):A5215.

24.Lee KH, et al. (2015)

Lee KH, Kim MJ, Lee JC, Lee JH, Lee JK. A case of solitary fibrous pleura tumor associated with severe hypoglycemia: Doege-Potter syndrome. Tuberc Respir Dis. 2015;78(1):120-124. (DOI: 10.4046/trd.2015.78.2.120)

25.Rocas D, et al. (2015)

Rocas D, et al. Solitary fibrous tumors of the pleura: not always a benign entity. Rev Port Pneumol. 2015;21(2):103-105. doi:10.1016/j.rppnen.2014.11.004.

26.Sawaga T, et al. (2017)

Sawaga T, Nagano T, Nakata M. Calcifying fibrous tumor disseminated on the pleura. Jpn J Chest Surg (J Jpn Assoc Chest Surg). 2017;31(4):265-269. doi:10.2995/jacsurg.31.265.

27.Edlin JC, et al. (2018)

Edlin JC, Donovan LE, Alexander C, Kanagasabay R. Recurrent pleural effusion in a young woman: calcifying fibrous tumour of the pleura. BMJ Case Rep. 2018;11(1):e226123. doi:10.1136/bcr-2018-226123.

28.Mazi A, et al. (2018)

Mazi A, Emil S, Bernard C, Canakis AM. Symptomatic calcifying fibrous tumor of the pleura in a teenager. J Pediatr Surg Case Rep. 2018;30:1-4. doi:10.1016/j.epsc.2017.10.011. PMCID: PMC5711393.

29.Lisowska H, et al. (2018)

Lisowska H, Marciniak M, Cianciara J, Pawełczyk K. A rare case of calcifying fibrous pseudotumor of the pleura with an accompanying vascular anomaly in the pulmonary ligament. Kardiochir Torakochirurgia Pol. 2018;15(1):59-61. doi:10.5114/kitp.2018.74678.

30.Mehrad M, et al. (2018) – Reference [7] in manuscript. Cases #32–34 from same article.Mehrad M, et al. [Complete citation not provided; please verify].

31.Massoth LR, et al. (2019)

Massoth LR, Selig MK, Little BP, Chebib I, Kradin RL. Multiple calcifying fibrous pseudotumors of the pleura: ultrastructural analysis provides insight on mechanism of dissemination.UltrastructPathol.2019;43(4-5):154-161.doi:10.1080/01913123.2019.1687631.

32.Bono GLP, et al. (2020)

Bono GLP, Lehner M, Schilling FH, Stahr N, Nowack M, Szavay PO. Complete resection of a large mediastinal calcifying fibrous tumor. Eur J Pediatr Surg Rep. 2020;8(1):e48-e51. doi:10.1055/s-0040-1713135.

33.Miyamoto N, et al. (2020)

Miyamoto N, Yoshida M, Tsuboi M, Kawakita N, Takizawa H, Kondo K, Tangoku A. A case of long-term unchanged calcifying fibrous tumor. Gen Thorac Cardiovasc Surg. 2020;68:1587-1590. doi:10.1007/s11748-020-01396-1.

34.Gorai K, et al. (2020)

Gorai K, Inoue T, Ohtsuka T. A case of calcifying fibrous tumor of the visceral pleura. J Jpn Assoc Chest Surg. 2020;34(5):398-402. doi:10.2995/jacsurg.34.398.

35.Hernandez Carrillo J, et al. (2021)

López‑Garnica D, Robles‑Pérez E, Peña‑Mirabal ES, Hernández‑Carrillo JA, Téllez‑Becerra JL. Tumor fibroso solitario gigante de la pleura [Giant solitary fibrous tumor of the pleura]. Cir Cir. 2010;78(1):31-43. Spanish. PMID: 20226113.

36.Jia X, et al. (2021)

Jia X, et al. Solitary fibrous tumors of the chest: an analysis of fifty patients. Front Surg. 2021;8:645318. doi:10.3389/fsurg.2021.645318.

37.Yokota R, et al. (2024)

Yokota R, Matsutani T, Mishima K, Yamagiwa R, Kubokura H, Motoda N, Taniai N, Yoshida H. Successful thoracoscopic enucleation of a calcifying fibrous tumor of the lower mediastinum in a young woman. Surg Case Rep. 2024;10(1):182. doi:10.1186/s40792-024-01981-z. PMID:39088123; PMCID: PMC11294290.

38.Shoji F, et al. (2024)

Noda N, Fukuyama S, Harada T, Takayama K, Shoji F, Yano T, et al. [Case of solitary fibrous tumor of the pleura completely resected eleven years after detection]. Nihon Kokyuki Gakkai Zasshi. 2009;47(5):421-6. (Article in Japanese, English abstract available). PMID: 19514506.

39.Our case (2025) – This is the current case report; no separate reference needed.
